# New Books

**Published:** 2008-01

Acid Rain in the Adirondacks: An Environmental History

Jerry Jenkins, Karen Roy, Charles Driscoll, Christopher Buerkett

Ithaca, NY:Cornell University Press, 2007. 256 pp. ISBN: 978-0-8014-4651-1, $65

Apoptosis and Cancer

Gil Mor, Ayesha Alvero, eds.

New York:Springer, 2008. 350 pp. ISBN: 978-1-58829-457-9, $99.50

Apoptosis, Senescence and Cancer, 2nd ed.

David A. Gewirtz, Shawn E. Holt, Steven Grant

Totowa, NJ:Humana Press, 2007. 975 pp. ISBN: 978-1-58829-527-9, $169

Applications of Toxicogenomic Technologies to Predictive Toxicology and Risk Assessment

Committee on Applications of Toxicogenomic Technologies to Predictive Toxicology and Risk Assessment, National Research Council

Washington, DC:National Academies Press, 2007. 278 pp. ISBN: 978-0-309-11298-7, $55

Autism and the Environment: Challenges and Opportunities for Research

Forum for Neuroscience and Nervous Systems Disorders

Washington, DC:National Academies Press, 2007. 364 pp. ISBN: 978-0-309-10881-2, $69.50

Cancer Genomics and Proteomics

Paul B. Fisher

Totowa, NJ:Humana Press, 2007. 481 pp. ISBN: 978-1-58829-504-0, $99.50

Chernobyl – What Have We Learned? The Successes and Failures to Mitigate Water Contamination Over 20 Years

Yasuo Onishi, Oleg V. Voitsekhovich, Mark J. Zheleznyak, eds.

New York:Springer, 2007. 289 pp. ISBN: 978-1-4020-5348-1, $129

Congenital Diseases and the Environment

P. Nicolopoulou-Stamati, L. Hens, C.V. Howard, eds.

New York:Springer, 2007. 471 pp. ISBN: 978-1-4020-4830-2, $199

Democratizing Technology: Risk, Responsibility and the Regulation of Chemicals

Anne Chapman

London, UK:Earthscan, 2007. 240 pp. ISBN: 978-1-8440-7421-1, $101.87

Exposed: The Toxic Chemistry of Everyday Products—Who’s At Risk and What’s At Stake for American Power

Mark Schapiro

White River Jct., VT:Chelsea Green Publishing, 2007. 216 pp. ISBN: 978-1-933392-15-8, $22.95

Increasing Capacity for Stewardship of Oceans and Coasts: A Priority for the 21st Century

Committee on International Capacity Building for the Protection and Sustainable Use of Oceans and Coasts, National Research Council

Washington, DC:National Academies Press, 2007. 150 pp. ISBN: 978-0-309-11376-2, $37

Microchip-Based Assay Systems

Pierre N. Floriano, ed.

New York:Humana Press, 2008. 275 pp. ISBN: 978-1-58829-588-0, $99.50

Monongah: The Tragic Story of the 1907 Monongah Mine Disaster, the Worst Industrial Accident in US History

Davitt McAteer

Morgantown, WV:West Virginia University Press, 2007. 332 pp. ISBN: 978-1-9332-0229-7, $30

Not One Drop: Betrayal and Courage in the Wake of the Exxon Valdez Oil Spill

Riki Ott

White River Jct., VT:Chelsea Green Publishing, 2008. 256 pp. ISBN: 978-1-933392-58-5, $21.95

Reproductive Health and the Environment

P. Nicolopoulou-Stamati, L. Hens, C.V. Howard, eds.

New York:Springer, 2007. 389 pp. ISBN: 978-1-4020-4828-9, $179

Risk Assessment of Chemicals: An Introduction

C.J. van Leeuwen, T.G. Vermeire, eds.

New York:Springer, 2007. 688 pp. ISBN: 978-1-4020-6101-1, $199

